# Utility of FDG PET/CT in the Management of Primary Testicular Lymphoma

**DOI:** 10.4274/mirt.14227

**Published:** 2018-06-07

**Authors:** Kürşat Okuyucu, Semra İnce, Engin Alagöz, Erman Ataş, Nuri Arslan

**Affiliations:** 1 University of Health Sciences, Gülhane Training and Research Hospital, Clinic of Nuclear Medicine, Ankara, Turkey; 2 University of Health Sciences, Gülhane Training and Research Hospital, Clinic of Pediatric Oncology, Ankara, Turkey

**Keywords:** Primary testicular lymphoma, SUVmax, FDG PET/CT

## Abstract

**Objective:**

Primary testicular lymphoma (PTL) is a form of extra-nodal lymphoma originating from the testicles. Currently, positron emission tomography (PET) with glucose analogue ^18^F-fluorodeoxyglucose (^18^F-FDG) is the most popular and widely used modality for evaluating tumor metabolism, and PTL usually displays increased ^18^F-FDG uptake. Despite the rapid increase in clinical applications of FDG PET/ computed tomography (CT), its role in PTL has neither been clearly defined nor reviewed systematically. This study reviews the usefulness and limitation of FDG PET/CT in the diagnosis and treatment of PTL.

**Methods:**

This study included 12 patients with PTL between 2004 and 2015. We retrospectively examined PET/CT results along with patient outcome. The maximum standardized uptake value (SUV_max_) was calculated.

**Results:**

The mean overall survival (OS) and disease-free survival (DFS) was 44.5 months and 35.5 months, respectively. The mean SUV_max_ was identified as 18.5 in recurrent/metastatic group. The 1-year and 3-year OS was 94% and 69%, while the 1-year and 2-year DFS was 93.5% and 56%, respectively.

**Conclusion:**

FDG PET/CT is very helpful in both staging and evaluating treatment response. Although it is not a perfect tool in the initial diagnosis, it might aid in the differential diagnosis of challenging testicular tumors. Pre-treatment and post-treatment FDG uptake values may also have a prognostic value in patients with PTL.

## Abstract (tr)

**Amaç:**

Primer testis lenfoması (PTL) testis kaynaklı bir ekstranodal lenfomadır. ^18^F-florodeoksiglukoz (^18^F-FDG) pozitron emisyon tomografisi/bilgisayarlı tomografi (PET/BT) tümör metabolizmasını değerlendirmede yaygın olarak kullanılmaktadır ve PTL yüksek FDG tutulumu gösterir. FDG PET/BT’nin klinik uygulamalarının gün geçtikçe artmasına rağmen, PTL’deki rolü tam olarak tanımlanmamış ve sistematize edilmemiştir. Bu çalışma FDG PET/BT’nin PTL tanı ve tedavisindeki yararlılık ve kısıtlamalarını araştırmaktadır.

**Yöntem:**

Çalışma 2004-2015 yılları arasında 12 PTL hastasını içermektedir. Olguların geriye dönük olarak FDG PET/BT sonuçları ve gidişatları incelenmiştir. Maksimum standart tutulum değeri (SUV_maks_) hesaplandı.

**Bulgular:**

Ortalama toplam sağkalım (OS) ve hastalıksız sağkalım (DFS) sırasıyla 44,5 ay ve 35,5 ay idi. Ortalama SUV_maks_ rekürrent/metastatik grupta 18,5 idi. OS 1. yılda %94, 3. yılda %69; DFS 1. yılda %93,5, 2. yılda %56 idi.

**Sonuç:**

FDG PET/BT evreleme ve tedaviye yanıt takibinde çok faydalıdır. Testis tümörlerinin ayırıcı tanısının zor olduğu olgularda yararlı olabilir. Fakat ilk tanıda iyi bir yöntem değildir. Tedavi öncesi veya sonrası FDG tutulum değerleri prognostik bir önem taşıyabilir.

## Introduction

Primary testicular lymphoma (PTL) is a form of extra-nodal lymphoma originating from the testicles (1). PTL constitutes approximately 1-2% of all non-Hodgkin lymphomas (NHL) and accounts for 1-9% of all testicular tumors (2). Most patients are older than 60 years of age, with PTL being the most frequent testicular neoplasm in this age group (3). Approximately 80-98% of PTLs are diffuse large B-cell lymphomas (DLBCL) (4,5). The typical initial symptom is a firm, painless testicular mass with an average tumor size of 6 cm (5). Synchronous bilateral involvement occurs in 6-10% while systemic disease is present in 20-30% of the patients (6). The median overall survival (OS) of PTL is reported to be 4-5 years (7). The most common metastatic sites are contralateral testicle, central nervous system (CNS), skin, adrenal glands, bone marrow, lung and pleura (8).

Diagnostic imaging modalities include ultrasound and magnetic resonance imaging (MRI), which allow simultaneous evaluation of both testicles, paratesticular space and spermatic cord (9). When PTL is suspected, inguinal orchiectomy is required for achievement of successful treatment and establishment of correct histopathologic diagnosis with adequate pathologic specimen. An experienced pathologist is required for difficult cases, since distinguishing some cases from seminoma can be challenging (10). In meta-analysis studies, 60-79% of the patients have stage I/II disease at initial presentation (11). Recommended staging is the same as in other forms of aggressive NHL by positron emission tomography/computed tomography (FDG PET/CT) and bone marrow biopsy, with the addition of specific CNS staging with lumbar puncture for cerebrospinal fluid analysis and cranial MRI (12). The most frequent metastatic location is CNS with a reported incidence of approximately 45% (13). 64% of CNS relapses involve the brain parenchyma (11).

Currently, PET with glucose analogue ^18^F-fluorodeoxyglucose (^18^F-FDG) is the most popular and widely used modality for evaluating tumor metabolism, and PTL usually displays increased ^18^F-FDG uptake (14). Despite the rapid increase in clinical applications of FDG PET/CT, its role in PTL has neither been clearly defined nor reviewed systematically. This study reviews the usefulness and limitation of FDG PET/CT in the diagnosis and treatment of PTL.

## Materials and Methods

This is a retrospective cohort study conducted with 12 PTL patients of DLBC variant treated by surgery (orchiectomy) and R-CHOP (rituximab, cyclophosphamide, doxorubicin, vincristine, prednisolone) between 2004 and 2015 at our institution. Patients with histopathologically confirmed PTL, who have been treated with R-CHOP protocol and had a follow-up of at least two years, with an initial staging or follow-up FDG PET/CT were included. Patients with systemic involvement at initial presentation and who received alternative therapy regimens other than R-CHOP as first line therapy protocol were excluded from the study. 

FDG PET/CT was obtained for primary staging in 8 cases and for evaluation of treatment response in 4 cases. Baseline staging, re-staging or follow-up PET results and patient outcome were retrospectively extracted from patient files. The patients having pathologically increased FDG uptake on baseline or follow-up FDG PET/CT constituted recurrent/metastatic group. The semi-quantitative parameter of maximum standardized uptake value (SUV_max_) was calculated on FDG PET/CT. OS was accepted as the time from initial diagnosis to death of any cause or last follow-up. Disease-free survival (DFS) was defined as the period from diagnosis to detection of first relapse or last follow-up.

### Ethics Committee Approval and Informed Consent

The study was approved by Gülhane Training and Research Hospital Institutional Ethics Committee (protocol number: 14044, date: 2014). Informed consent was obtained from all participants.

### FDG PET/CT Imaging Protocol

Patients fasted for at least 6 hours and their blood glucose level were below 150 mg/dL before the injection of an activity of 370-555 MBq of ^18^F-FDG calculated according to the patient’s bodyweight. Images were acquired one hour later with an integrated PET/CT scanner (Discovery 690-GE Healthcare). Unenhanced low dose CT and PET emission data were performed from mid-thigh to the vertex of the skull in the supine position with the arms raised above the head. CT data was obtained by an automated dose modulation of 120 kVp (maximal 100 mA), a collimation of 64×0.625 mm, a measured field of view (FOV) of 50 cm, and a noise index of 20%. These data were reconstructed to images of 0.625 mm transverse pixel size and 3.75 mm slice-thickness. PET data was in 3D mode with a scan duration of 2 min per bed position and an axial FOV of 153 mm. A standard technique (random, scatter and attenuation) and an iterative reconstruction (matrix size 256×256, Fourier rebinning, VUE Point FX [3D] with 3 iterations, 18 subsets) corrected the emission data.

### Visual and Quantitative Assessment of FDG PET/CT

A standard protocol on a dedicated workstation (Volumetrix for PET/CT and AW volume share 4.5, GE Healthcare, Waukesha, WI, USA) calculated the semiquantitative PET/CT parameters used in the study. The SUV_max_ corrected for body weight was computed with standard methods from the activity of the highest density voxel in three-dimensional tumor region of transaxial whole body images on attenuation-corrected PET/CT images. The corresponding CT scan of lesions were demarcated as a frame if the boundaries of an uptake were difficult to define for the calculation of SUV_max_.

### Statistical Analysis

The data were analyzed by IBM Corp. Released 2013 (IBM SPSS Statistics for Windows, Version 22.0. Armonk, NY: IBM Corp.) number and percentage values were used for the description of categorical data; mean, median, standard deviation (SD), minimum (min) and maximum (max) values were used for the description of continuous data. ROC curve was drawn to evaluate the diagnostic accuracy of SUV_max_ and a cut-off level was determined. Sensitivity and specificity rates were calculated according to the chosen cut-off value. Kaplan-Meier test was used for survival analysis.

## Results

The mean patient age was 57±15 years (21-77) and the mean SUV_max_ value was 18.5±7 (9.8-30.8). Mean OS and DFS were identified as 44.5 months (12-101) and 35.5 months (9-101), respectively. Mean SUV_max_ was 15.9 in the recurrent/metastatic group. Complete remission was achieved in 5/12 of the cases (42%). On the other hand, 7/12 (58%) developed recurrence and/or metastasis during follow-up 5 (42%) of which died. Median time to progression was 18.6 months. OS at the 1^st^ year was 94%, 87.5% at the 2^nd^, 69% at the 3^rd^, and 62.5% at the 5^th ^years. DFS at the first year was 93.5%, 56% at the second year, and 44% at the 3^rd^ year. 

**Figure 1:**
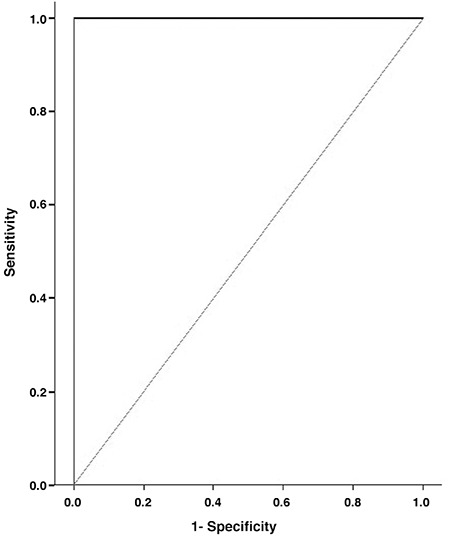
ROC curve represents the diagnostic accuracy of SUV_max_

**Figure 2:**
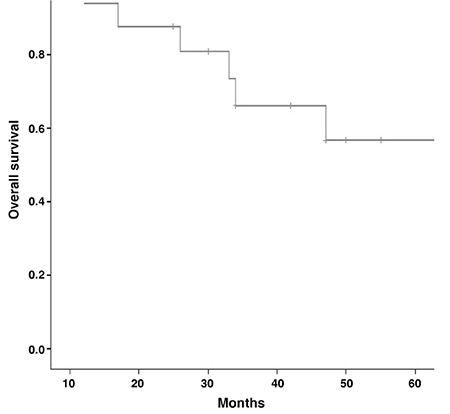
Kaplan-Meier curve shows the plot associated with overall survival

**Figure 3:**
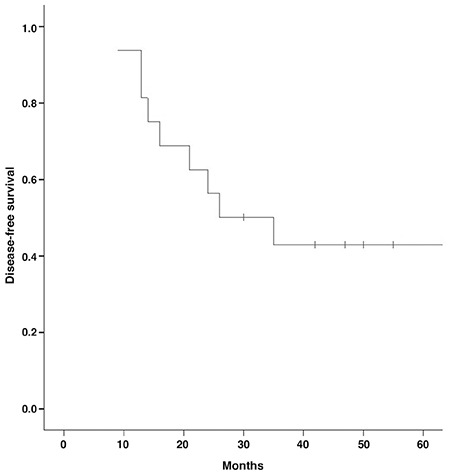
A Kaplan-Meier curve indicates disease-free survival in patients with primary testicular lymphoma

There was a statistically significant difference between recurrent/metastatic group and non-metastatic (complete remission) group according to SUV_max_ values (p<0.001). ROC curve was drawn to evaluate the diagnostic value of SUV_max_ (Figure 1). Cut-off value of SUV_max_, its associated sensitivity and specificity rates are demonstrated in Table 1. Kaplan-Meier method was used to compare DFS and OS of recurrent/metastatic and non-metastatic groups, and curves were plotted for OS (Figure 2) and DFS (Figure 3).

## Discussion

The mean age of our patient population was 57 years, which is lower than the mean age reported in the literature. The mean tumor size was 6.5 cm and the mean SUV_max_ was 18,5, which were both in line with previous studies. Local recurrence was detected in 60% of the cases and bilateral involvement was present in 16%. Nearly 50% of the tumors metastasized to CNS, and CNS metastasis and/or testicular recurrence was the cause of death in all our cases. All these findings including median OS, median time to progression and other prognostic data presented herein are in accordance with the literature.

Early diagnosis of PTL is essential for timely treatment before the occurrence of widespread metastases and invasion of neighboring tissues. Clinical diagnosis of PTL is sometimes difficult and delayed due to its nonspecific symptoms. FDG PET/CT has a limited value in the initial diagnosis and differentiation of PTL from other malignant testicular tumors or non-malignant lesions. PTL is a highly cellular tumor and shows increased glucose metabolism, causing homogenous marked asymmetric testicular FDG uptake (14). The semi-quantitative FDG uptake values measured by SUV_max_ were reported to be between 10 and 30 for PTL (14). This value is higher than the average SUV_max_ of the normal testicle that has been reported as approximately 2.5 in large case series (14). PTL may be distinguished from other testicular diseases such as seminoma according to its uptake pattern and SUV_max_ value. However, FDG PET/CT is not a perfect tool in the diagnosis of PTL. 

Detection of PTL with extra-testicular involvement at primary (baseline) staging is important for appropriate therapy since systemic involvement alternates therapy protocol. Standard baseline (initial) staging for PTL includes MRI and sometimes bone marrow biopsy to exclude systemic lymphoma (13). Conventional initial staging in PTL might not show extra-testicular lesions, especially occult systemic lymphoma in some patients. FDG PET/CT is more sensitive than conventional staging methods in this regard and may disclose higher rates of concomitant systemic disease at initial diagnosis. The supremacy of this modality is attributed to the ability to screen the whole-body, and the fusion of anatomic detail provided by the CT component with metabolic information. This advantage remains true also for re-staging during follow-up. The clinical significance of identifying recurrence and/or metastasis at the follow-up is apparent. Currently, FDG PET/CT is the choice of imaging modality for primary staging and re-staging. In our study, we also observed that FDG PET/CT is an excellent tool in disease-staging both initially and during follow-up.

PTL is one of the most malignant testicular tumors that respond well to treatment. Changes in metabolic imaging with FDG PET/CT can be detected soon after initiation of therapy. Early evaluation of the initial treatment response is very important, since salvage treatment may improve outcome. Follow-up (evaluation of treatment response) FDG PET/CT fulfills this easily with its unique advantages over conventional techniques. The whole body can be assessed in one session prior to anatomic changes and active foci can be distinguished from fibrotic or inflammatory tissue as well as detection of small lesions located outside areas imaged by conventional techniques. Dramatic disappearance of increased FDG uptake in the tumor with therapy indicates treatment success. 

Kawai et al. (15) demonstrated that FDG PET 3 weeks after the first chemotherapy showed a significant decrease in FDG uptake of the tumor as compared to pre-treatment uptake. The reduction in FDG uptake significantly correlated with the decrease in tumor size as detected by follow-up MRI (15). These results indicate that metabolic imaging with FDG PET can accurately evaluate treatment response at an early stage, sometimes preceding changes on MRI. Early therapeutic monitoring might have an impact on deciding whether the treatment regimen should be maintained or changed. If patients with a poor early response were identified, treatment could be modified at an early stage before delivering additional cycles of ineffective therapy. In our study, we detected that follow-up FDG PET provided valuable information for treatment evaluation.

The 5-year survival rate is reported as 35% with a mean survival of 13 months for patients with PTL (16). The 5-year survival rate was identified as 53% in our study, with a mean survival of 18 months. Early quantitative measurement of metabolic response with FDG PET was declared to provide valuable prognostic information in systemic aggressive lymphoma types (15). FDG PET/CT might also provide prognostic information in patients still under therapy and predict long-term outcome in PTL. The OS of patients with low to moderate FDG uptake was reported to be significantly longer than that of patients with high FDG uptake (17). PTL with high FDG uptake tended to exhibit poor treatment response as compared to that with low to moderate uptake (18). This prognostic information yielded by FDG PET over SUV_max_ values was very accentuated in our study. The SUV_max_ of the recurrent/metastatic group was significantly high (p<0.001) and it was a strong predictor of outcome. To the best of our knowledge, although there are no published studies on this issue in the literature, FDG uptake value might represent tumor aggressiveness in PTL. Further clinical trials are required to define the optimal strategy to utilize FDG PET information as a prognosticator and to improve outcome in patients with PTL.

## Conclusion

The utility of FDG PET/CT is currently increasing in the management of PTL. This review summarizes the usefulness and limitations of FDG PET/CT in the diagnosis and treatment of PTL. FDG PET/CT is valuable in both primary staging and restaging. It is also valuable in evaluating treatment response after initial chemotherapy and altering treatment strategy at a very early stage, if required. Pre- and post-treatment FDG uptake values reflected by SUV_max_ might have a prognostic value in patients with PTL. FDG PET/CT might be useful for differential diagnosis of challenging testicular tumors. Nevertheless, it is not the proper modality for initial diagnosis.

## Figures and Tables

**Table 1:**
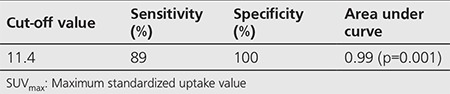
Cut-off value of SUV_max_, its associated sensitivity and specificity are demonstrated
